# Investigation of the photoluminescent properties, scintillation behaviour and toxicological profile of various magnesium tungstate nanoscale motifs

**DOI:** 10.1098/rsos.220994

**Published:** 2022-12-07

**Authors:** Nathaniel Hurley, Sailesh Srinivas, Justin Fang, Manli Sun, Simon Hong, Chia Te Chien, Alan Guo, Tamor A. Khan, Mingxing Li, Mircea Cotlet, Federico Moretti, Edith Bourret, Daniel Radin, Stella E. Tsirka, Maya Shelly, Stanislaus S. Wong

**Affiliations:** ^1^ Department of Chemistry, State University of New York at Stony Brook, Stony Brook, NY 11794-3400, USA; ^2^ Department of Neurobiology, State University of New York at Stony Brook, Stony Brook, NY 11794-5230, USA; ^3^ Center for Functional Nanomaterials, Brookhaven National Laboratory, Building 735, Upton, NY 11973, USA; ^4^ Materials Sciences Division, Lawrence Berkeley National Laboratory, Berkeley, CA 94720, USA; ^5^ Department of Pharmacological Sciences, State University of New York at Stony Brook, Stony Brook, NY 11794-8651, USA

**Keywords:** nanomaterials, scintillation, toxicology, bioimaging, synthesis, oxides

## Abstract

We have synthesized several morphologies and crystal structures of MgWO_4_ using a one-pot hydrothermal method, producing not only monoclinic stars and large nanoparticles but also triclinic wool balls and sub-10 nm nanoparticles. Herein we describe the importance of reaction parameters in demonstrating morphology control of as-prepared MgWO_4_. Moreover, we correlate structure and composition with the resulting photoluminescence and radioluminescence properties. Specifically, triclinic-phase samples yielded a photoluminescence emission of 421 nm, whereas monoclinic-phase materials gave rise to an emission maximum of 515 nm. The corresponding radioluminescence data were characterized by a broad emission peak, located at 500 nm for all samples. Annealing the wool balls and sub-10 nm particles to transform the crystal structure from a triclinic to a monoclinic phase yielded a radioluminescence (RL) emission signal that was two orders of magnitude greater than that of their unannealed counterparts. Finally, to confirm the practical utility of these materials for biomedical applications, a series of sub-10 nm particles, including as-prepared and annealed samples, were functionalized with biocompatible PEG molecules, and subsequently were found to be readily taken up by various cell lines as well as primary cultured hippocampal neurons with low levels of toxicity, thereby highlighting for the first time the potential of this particular class of metal oxides as viable and readily generated platforms for a range of biomedical applications.

## 1. Introduction

Tungstates denote a class of materials with the general formula of AWO_4_ (A = divalent metal cation). Most tungstates, with the exceptions of HgWO_4_ and SnWO_4_, crystallize in either the wolframite or scheelite structure [1]. Wolframite structures are characterized by a monoclinic crystal structure with octahedral sites, while scheelites adopt a tetragonal crystal structure with tetrahedral sites [2]. Whether a tungstate crystallizes in either the wolframite or scheelite arrangement is dependent upon the ionic radius of the A^2+^ cation in which ionic radii greater than 0.99 Å (Ca, Sr, Ba and Pb) yield the scheelite structure, whereas ionic radii less than 0.97 Å (Cd, Zn, Mn, Mg and Fe) give rise to the wolframite structure [2]. Of specific interest to us herein, MgWO_4_ maintains an ionic radius less than 0.97 Å, possesses a monoclinic wolframite configuration, and belongs to the P2/c space group [3]. However, the crystal structure of MgWO_4_ varies with temperature; its tetragonal form is predominant until a temperature of approximately 800–850°C is achieved, whereby it transforms into its monoclinic phase. Subsequent heating above 1165°C yields a triclinic arrangement [4].

There have been a number of synthetic routes reported for the preparation of MgWO_4_ nanostructures, including but not limited to the hydrothermal/solvothermal method, microwave-assisted reactions, the solid-state process and flux growth techniques, in addition to electrospinning and calcination-based protocols [4–12]. Use of these different procedures has led to the generation of various discrete and distinctive shapes, including nanowires [11], irregular nanoparticles[13,14], bulk formulations [4,10,15], nanosheets [6,8], nanoparticles [16], nanorods [3,8,17], wool balls [8,16] and stars [8,9]. In addition, it has been reported that both the monoclinic wolframite and the triclinic crystal phases of these metal tungstates can be produced, most frequently under high-temperature annealing conditions [6,8,11,13].

Based on this set of aforementioned synthetic methods, we chose to employ a facile hydrothermal method, which is advantageous in terms of its relative simplicity of operation, high yield, large volume, low cost, and synthetic adaptability in terms of yielding a number of motifs, characterized by monodisperse size and shape [8]. The hydrothermal process involves the sealing of a precursor solution in a Teflon-lined vessel within a steel-jacketed autoclave followed by heating in an oven. The high-pressure and high-temperature environment created by the hydrothermal method can foster the growth of crystalline products.

Because of its inherent flexibility, the hydrothermal synthesis process can be used to fabricate MgWO_4_ nanostructures with reliable shape control. Indeed, the ability to tailor and tune the shape and crystal structure of tungstates has implications for its optoelectronic behaviour. For instance, the photoluminescent properties of MgWO_4_ have been noted to vary as the crystal structure shifts from the triclinic to the monoclinic phase. The triclinic phase emits at approximately 430 nm, a phenomenon attributed not only to the presence of surface defects and vacancies but also to the amorphous nature of the MgWO_4_ framework. By contrast, the corresponding monoclinic phase after post-annealing emits at approximately 470 nm, an emission signal which can be ascribed to the radiative electronic transitions within the (WO_6_)^6−^ complex. This optical behaviour was observed in both nanoparticles and nanowires [11,13,18].

Ce-doped MgWO_4_ possessed a similar emission maximum at 470 nm within a range of 400 to 550 nm. It was found that increasing the amount of Ce ion dopant content to 3% yielded a higher emission intensity [19]. In addition, similar types of tungstates, including CdWO_4_, ZnWO_4_, CoWO_4_ and FeWO_4_, characterized by the monoclinic crystal phase, have evinced similar emission maxima, situated in the range of 460 to 470 nm [20–23]. Whereas many of these aforementioned studies have focused on a single morphology, few reports have systematically analysed PL properties as a function of varying particle morphology and/or crystal structure.

As a crucial and relevant opto-electronic application, scintillators can function as energy transducers by absorbing high-energy radiation, such as X-rays, and subsequently, luminescing in response, thereby emitting lower energy light, such as UV or visible light in the process. This useful luminescence response has rendered scintillators not only as excellent detectors of high-energy radiation but also as intermediary bridging species capable of spanning the wide energy gap between X-rays and typical photosensitizing agents. That is, upon X-ray irradiation, the scintillator can absorb and then emit either lower energy UV or visible light, and thereby excite the photosensitizing species. This paradigm is critical for enabling efficient energy transfer between the scintillator and the photosensitizer, a process which underlies the effective generation of reactive oxygen species associated with successful photodynamic therapy (PDT). PDT represents a desirable cancer treatment method, because it has been shown to be minimally invasive, has a lower risk of systemic toxicity, and is highly specific for targeting tumors [24,25]. X-ray-activated scintillators can maintain a virtually unlimited tissue penetration depth, especially as compared with either conventional UV or visible light irradiation [26]. As such, the key to developing MgWO_4_ as a viable and biologically relevant enabler of PDT is (i) to highlight its photoluminescence and radioluminescence performance, and (ii) to simultaneously demonstrate its comparatively low toxicity under biologically relevant conditions [27].

As a corollary benefit, it is worth noting that an X-ray-activated MgWO_4_ scintillator can also serve as an equally effective bio-imaging agent. Traditional organic molecules and quantum dots, normally tasked for these imaging purposes, are known to suffer from photobleaching and toxicity effects. Moreover, they are impractical for a number of applications, due to the limited penetration depth of IR/visible/UV irradiation, which is necessary to activate them. In principle, the use of scintillating nanoparticles such as MgWO_4_ can overcome these issues as they are inherently less susceptible to photobleaching, thereby rendering them as an attractive, alternative option to conventional labelling methods [28].

Wide bandgap semiconductors and insulators can function as fast and luminous classes of scintillating materials [8]. A key characteristic of a high-performing scintillator is the incorporation of a high atomic number species (such as W), which translates to greater stopping power, i.e. the amount of energy dissipated within a material by ionizing radiation [8]. As relevant examples of interest herein, materials, such as CdWO_4_, CaWO_4_, SnWO_4_ [29,30] and Bi_2_WO_6_ [31], upon high-energy irradiation, represent a set of promising tungstate-based scintillators [5,17]. Previous studies on the scintillating properties of MgWO_4_ itself have found that it maintains a high scintillation output and emits at 470 nm with a high radioluminescence intensity equivalent to 35% of CdWO_4_, coupled with a decay time of 36 µs for the emitted light [5]. It is expected that the scintillating property of MgWO_4_ may vary as a function of size, shape and crystal structure, but this issue has not yet been fully explored. Addressing this problem is a key objective of our current investigation.

The first part of our study, therefore, highlights our efforts to enable effective simultaneous size, shape and composition control of MgWO_4_ nanostructures, based on the tuning of existing protocols in the literature. This work included the optimization of discrete reaction parameters to yield monodisperse and homogeneous families of MgWO_4_ nanostructures. The optimized reaction variables included solvent, reaction temperature, reaction time, use of surfactants and pH. Samples were subjected to structural and chemical characterization, via X-ray diffraction (XRD), scanning electron microscopy (SEM) and transmission electron microscopy (TEM). Furthermore, we have probed optical properties of these materials in the context of both (i) scintillation and (ii) photoluminescence. Finally, based on these favourable optical attributes, we have theorized on the potential (iii) viability and biocompatibility of these materials as labels and platforms for biomedical applications. We should note that a proper demonstration of the use of MgWO_4_ in PDT and *in vivo* biological labelling is well beyond the scope of the current study. What we present instead are arguments and data for the potential inclusion of this material as a candidate for such an important role and purpose.

## 2. Experimental section

### 2.1. Materials

All chemicals were used, without additional purification. Sodium tungstate dihydrate (Na_2_WO_4_ · 2H_2_O, 99.0+%) and ethanol (EtOH, 90% denatured) were procured from Beantown Chemical. Magnesium nitrate hexahydrate (Mg(NO_3_)_2_ · 6H_2_O, 99.9%) was purchased from Mallinckrodt Chemical Works. Ethylene glycol (EG, 99.0+%), Triton X-114 (TX-114, reagent grade) and sodium dodecyl sulfate (SDS, 99+%) were acquired from VWR Life Science. Polyethylene glycol (PEG, M.W. of 4000 g mol^−1^), hexadecyltrimethylammonium bromide (CTAB, 98+%), poly-l-ornithine (PLO) and paraformaldehyde (PFA, 95%) were purchased from Sigma Aldrich. Triethylene glycol (TEG) was sourced from Kodak Chemicals. Absolute ethanol was purchased from Pharmco. Methanol, hydrochloric acid (HCl, 36.5–38%) and polyvinylpyrrolidone (PVP, M.W. of 40 000 g mol^−1^) were all bought from Alfa Aesar. Sodium hydroxide (NaOH, 98.5%) and (3-aminopropyl)triethoxysilane (APTES, C_9_H_23_NO_3_Si, 99%) were obtained from Acros Organics.

Fluorescein 5-isothiocyanate (FITC) was bought from Thermo Fisher Scientific. Biological reagents, including Dulbecco's Modified Eagle Medium (DMEM), FluoroBrite medium, Neurobasal medium, fetal bovine serum (FBS), 0.5% trypsin-EDTA, B-27 neural cell culture supplement, Glutamax supplement and penicillin/streptavidin (10 000 units ml^−1^), were all gathered from Gibco, Inc. Phosphate buffered saline (PBS) and molecular biology grade water were obtained from Corning, whereas DAPI Fluoromount-G was acquired from Southern Biotech. Deionized (DI) water was collected from our laboratory itself, whereas ultrapure water came from our Milli-Q 7000 ultrapure water system.

### 2.2. Synthesis

#### 2.2.1. General reaction protocol for synthesis of magnesium tungstate (MgWO_4_)

The generalized syntheses of different magnesium tungstate (MgWO_4_) morphologies were carried out via a modified facile hydrothermal method [8]. Depending on the targeted shape, we tweaked the experimental reaction conditions, so as to achieve the desired synthetic objective. Precursor stock solutions were prepared by dissolving 3.298 g of Na_2_WO_4_ · 2H_2_O (0.01 mol) and 2.564 g of Mg(NO_3_)_2_ · 6H_2_O (0.01 mol) in separate vials with 10 ml of deionized (DI) water. Then 0.5 ml of each individual precursor solution was added to a 23 ml Teflon-lined vessel. To create the various observed morphologies, different surfactants and ethylene glycol/water solvent ratios were used. Solutions were stirred for 30 min. After the stirring process, aliquots of either 1 M or 5 M aqueous NaOH were used to adjust the solution pH to 10.3. The solution within the Teflon-lined vessel was heated to a temperature of 220°C for 48 h. The resulting precipitate was subsequently washed three times with water and then twice with ethanol at 9000 r.p.m. for 5 min per wash. The final solution was dispersed in 5 ml of ethanol.

The balanced equation for the overall reaction is represented below, as per equation (2.1).

2.1 $${\mathrm{Na}}_{2}{\mathrm{WO}}_{4}(aq)+\mathrm{Mg}{\left(NO_{3}\right)}_{2}(aq)\underset{\mathrm{high}\;\mathrm{temperature}\;\mathrm{and}\;\mathrm{pressure}\;\mathrm{hydrothermal}\;\mathrm{reaction}}{\to }2\mathrm{NaN}O_{3}(aq)+{\mathrm{MgWO}}_{4}(s).$$

#### 2.2.2. Synthesis of sub-10 nm MgWO_4_ nanoparticles

For the production of these ultra-small MgWO_4_ nanoparticles, 16.5 ml of ethylene glycol was used as the solvent and 0.168 g PVP was introduced as a surfactant, so as to stabilize the particle growth. The sample was then added to a Teflon-lined autoclave, stirred for 30 min, and finally processed hydrothermally, as indicated above.

#### 2.2.3. Synthesis of MgWO_4_ wool balls

For the reliable generation of MgWO_4_ wool balls, a mixture of 7.5 ml of ethylene glycol and 9 ml DI water was used as the solvent in the absence of surfactant. The sample was then inserted into a Teflon-lined autoclave, stirred for 30 min and ultimately heated hydrothermally, as previously described.

#### 2.2.4. Synthesis of larger MgWO_4_ nanoparticles

For the synthesis of relatively larger MgWO_4_ nanoparticles, 16.5 ml of ethylene glycol was used as the solvent and 0.168 g PVP was introduced as the surfactant with which to stabilize the particle growth. The sample was then added to a Teflon-lined autoclave and stirred for 30 min. After the stirring step, the pH was increased to 10.3, using an aqueous 5 M NaOH solution that had been added in dropwise. The solution was then allowed to stir for 10 min, once the desired pH had been attained. After the pH adjustment, the sample was heated hydrothermally and then washed, as discussed earlier.

#### 2.2.5. Synthesis of MgWO_4_ stars

For the fabrication of MgWO_4_ stars, a mixture of 8.25 ml of ethylene glycol and 8.25 ml DI water was used as the solvent along with 0.192 g PEG as the surfactant to stabilize particle growth. The sample was then added to a Teflon-lined autoclave with stirring for 30 mins. After the stirring step, the pH was increased to 10.3 using an aqueous 5 M NaOH solution that had been added dropwise. The resulting solution was allowed to stir for 10 min, once the targeted pH had been reached. After the pH modification, the sample was heated hydrothermally and subsequently washed, as explained above.

#### 2.2.6. Annealing method

To modify the crystal structure of the sub-10 nm particles and corresponding wool balls, we used an annealing method. Specifically, we heated as-synthesized morphologies, described in §§2.2.2 and 2.2.3, within a tube furnace at 800°C for 3 h and at a ramp rate of 10°C min^−1^. Samples were collected and processed without either further washing or purification.

#### 2.2.7. Functionalization of sub-10 nm particles with (3-aminopropyl)triethoxysilane

Sub-10 nm MgWO_4_ particles were coated with (3-aminopropyl)triethoxysilane (APTES) by suspending 50 mg of particles in 10 ml of ultrapure water. Then, 0.14 ml of APTES was added to 5 ml of absolute ethanol and combined with the MgWO_4_ solution. The pH of the solution was adjusted to 11 using 5 M NaOH, and it was heated to 50°C for 5 h while stirring. The particles were collected by centrifugation, washed three times with ultrapure water, and resuspended in 10 ml ultrapure water to produce chemically derivatized MgWO_4_-APTES.

#### 2.2.8. Labelling with fluorescein isothiocyanate

One milligram of fluorescein isothiocyanate (FITC) was dissolved in 5 ml of methanol and added to the MgWO_4_-APTES medium. The solution was stirred for 3 h in an environment devoid of light after which the particles were collected by centrifugation, washed five times with ultrapure water, and resuspended in 10 ml of ultrapure water to produce MgWO_4_-APTES-FITC samples.

#### 2.2.9. Coating with polyethylene glycol

Two hundred milligrams of biocompatible PEG were dissolved in 4 ml of water and added to the MgWO_4_-APTES-FITC solution. The mixture was stirred overnight and protected from light, after which the particles were collected by centrifugation and resuspended in 10 ml of ultrapure water to produce MgWO_4_-APTES-FITC-PEG.

### 2.3. Cell line culture

Two cell lines were chosen for testing, including COS-7 [32] and HEK-293 [33]. In particular, COS-7 cells are derived from African green monkey kidney cells which had been transformed with a modified SV40 virus. This procedure allows them to be readily transfected with DNA in its plasmid form and facilitates expression of that DNA. The ability to easily modify COS-7 cells in order to produce arbitrary proteins of interest has led to their wide applicability in biomedical research. Separately, HEK-293 cells are denoted as human embryonic kidney cells, which were transformed and immortalized with the AD5 adenovirus. As with COS-7, these cells can be facilely transfected with plasmids in order to express targeted genes of interest, and not surprisingly, are also commonly used in biomedical research.

#### 2.3.1. Seeding of the cell line

COS-7 and HEK-293 cells were grown in specialized media, incorporating Dulbecco's Modified Eagle Medium (DMEM) with 10% fetal bovine serum (FBS) and 1% penicillin/streptavidin (10 000 units ml^−1^) within 100 mm Petri dishes in a Heracell 150i incubator (Thermo Fisher) at 37°C and 5% CO_2_. For plating for particle uptake experiments, the medium was removed, and the cells were collected by trypsinization (0.5% trypsin-EDTA diluted 10× with DMEM). The cells were resuspended in media and plated on 12 mm glass coverslips at a density of approximately 35 000 to 60 000 cells per coverslip in a series of 24-well culture dishes. The coverslips were coated with poly-L-ornithine (PLO; prepared in water at 100 µg ml^−1^) and rinsed with molecular biology grade water, before cell plating. The cells were later incubated for 24 h at 37°C, prior to particle application.

#### 2.3.2. Particle application

The cells either were applied with 20 µg of MgWO_4_-APTES-FITC-PEG or left untreated with particles, with the latter serving as a control sample. Following particle application, the cells were incubated for time periods ranging between 2 and 24 h at 37°C, prior to fixation.

#### 2.3.3. Fixing cells

The growth media were removed, and the cells were washed twice with phosphate-buffered saline (PBS). The cells were fixed in 0.5 ml of 4% paraformaldehyde (PFA) for 15 min, followed by extensive washes with 1 ml of PBS. The coverslips were then mounted onto glass slides with DAPI Fluoromount-G to allow for staining of cell nuclei.

#### 2.3.4. Cell viability

Cell viability was assayed using an MTT assay kit (Vybrant MTT Cell Proliferation Assay Kit, Molecular Probes), according to the method, described as follows. Fifty microlitres of poly-L-ornithine (PLO) in water at 100 µg ml^−1^ was added to each well of a 96-well plate, and the plate was incubated overnight at 37°C to coat the wells with PLO, before removing the PLO solution and rinsing twice with 100 µl of molecular biology grade water. Cells were cultured and suspended, as discussed in §2.3.1. Then 2 ml of the cell suspension was diluted to 10 ml with media; 100 µl of this diluted suspension was added to the wells of the PLO-treated 96-well plate and incubated at 37°C overnight. Subsequently, either as-prepared or annealed MgWO_4_ particles, coated with APTES and PEG, were added in either low (2 µg ml^−1^), medium (20 µg ml^−1^) or high (200 µg ml^−1^) concentrations, respectively, and incubated in the culture medium for 24 h at 37°C. Five milligrams of MTT (3-(4,5-dimethylthiazol-2-yl)-2,5-diphenyltetrazolium bromide) was dissolved in 1 ml PBS to form an MTT stock solution. The cell culture medium was then removed and subsequently replaced with 100 µl of Fluorobrite DMEM media containing 10 µl MTT stock solution. The cells were next incubated for 4 h at 37°C. One gram of sodium dodecyl sulfate (SDS) was dissolved in 10 ml of 0.01 N HCl, and 100 µl of this solution was added to each well. The plate was consequently incubated for 18 h at 37°C. The absorbance of each well was measured at 570 nm using a plate reader and compared with that of a standard curve, prepared by serial dilutions of the original cell suspension.

#### 2.3.5. Neuronal culturing and nanoparticle treatment

Coverslips initially coated with PLO (as per §2.3.1) were further coated with laminin by incubating overnight in 0.5 ml of laminin solution (5 µg ml^−1^ in Neurobasal media). Dissociated E18 embryonic rat hippocampal neurons were seeded at a density of approximately 20 000–50 000 cells per well and incubated for 2 days at 37°C [34], prior to treatment with 10 µg of particles per well for 20 h. The cells were subsequently fixed, as discussed in §2.3.3 for confocal imaging. Primary dissociated hippocampal neuronal cultures were prepared from embryonic rats, according to protocols approved by the Institutional Animal Care and Use Committees at Stony Brook University.

#### 2.4. Characterization

#### 2.4.1. X-ray diffraction

XRD patterns were used to collect information regarding the crystal structure and chemical composition of the as-obtained MgWO_4_ product. The measurements were carried out on a Rigaku MiniFlex 600 X-ray diffractometer. The instrument was operated at 40 kV and 15 mA, using Cu-K*_α_* radiation (*λ* = 1.5406 Å). The samples were prepared for powder XRD by sonicating the MgWO_4_ powder, dispersed in 5 ml of ethanol. Aliquots of the sample were then drop cast onto a silica zero diffraction plate for XRD (MTI Corporation, p-type silicon and B-doped, with a 23.6 mm diameter and a 2 mm thickness). Measurements were collected from 2*θ* values of 10°–90° at a rate of 10° min^−1^, using a scan step of 0.02° per scan. XRD patterns were analysed using the Match! program with the monoclinic standard pattern of this material used as a baseline for comparison.

#### 2.4.2. Scanning electron microscopy

SEM was used to image most of the samples of MgWO_4_ in order to analyse morphology and to obtain quantitative measurements of size. SEM images were collected with the JEOL JSM-7600F scanning electron microscope at accelerating voltages between 5 and 10 kV. Samples were prepared for SEM imaging by sonicating the MgWO_4_, dispersed in 5 ml of ethanol, and then drop-casting aliquots onto silica wafers.

#### 2.4.3. Transmission electron microscopy

TEM images were collected with the JEOL 1400 transmission electron microscope at accelerating voltages between 80 and 120 kV. The instrument was also equipped with a 2048 × 2048 Gatan charged-coupled device (CCD) camera. MgWO_4_ samples were prepared for TEM imaging by sonicating the powders dispersed in ethanol and drop casting aliquots onto copper grids which were coated with lacey carbon. Both SEM and TEM images were quantitatively analysed with the use of the ImageJ software to record measurements of average size and standard deviation.

#### 2.4.4. Photoluminescence

PL spectra and time-resolved PL decays were recorded by an Ocean Optics FL65000 fibre optics spectrometer and a time-correlated single-photon counting (TCSPC) technique with a femtosecond laser as the excitation source. PL decays were recorded using a 20 nm bandpass filter and an excitation frequency of 10 kHz. Decays were individually fit with FluoFit PicoQuant software using a multi-exponential fitting model. A PL excitation source centred at 280 and 340 nm was used for the analysis of the monoclinic and triclinic phases of the materials, respectively. Decay times were acquired at the wavelengths, characterized by the highest PL signal. Samples were measured by placing dry powder between two quartz slides. The slides were then taped at the edges to prevent the sample from moving and placed within the beam to initiate the measurements.

PL spectra and excitation spectra were also obtained with the same light detection set-up used for radioluminescence (§2.4.5). Excitation was performed using a 75 W Xe lamp coupled to a monochromator. Both the excitation and emission spectra were corrected for the experimental response of the system.

#### 2.4.5. Radioluminescence

Radioluminescence (RL) spectra were detected using a Princeton CCD and monochromator set-up with the excitation conditions determined to be 50 kV and 50 mA, using a copper rotating anode X-ray generator (Brüker). All of the spectra are corrected for the emission response of the apparatus. Excitation spectra were also corrected for lamp intensity.

Pulsed X-ray decays have been obtained with the TCSPC technique using a custom-built system, composed of an ultrafast Ti:sapphire laser, a light-excited X-ray tube (Hamamatsu N5084) and a multi-channel plate photomultiplier (Hamamatsu R3809U-50). The ‘start and stop’ signal timing was obtained with an Ortec 9308 ps analyser. No spectral selection was used in the collection of the decay signals.

#### 2.4.6. Confocal imaging

The fixed cells were imaged with an Olympus FV1000 laser-scanning confocal microscope with an oil-immersion 40X objective. A 405 nm laser and a 425–475 nm detector were used for DAPI, whereas a 488 nm laser and a 500–600 nm detector were utilized for FITC. Images were collected and processed using the intrinsic Olympus FV10-ASW software.

## 3. Results and discussion

**Figure 1.**
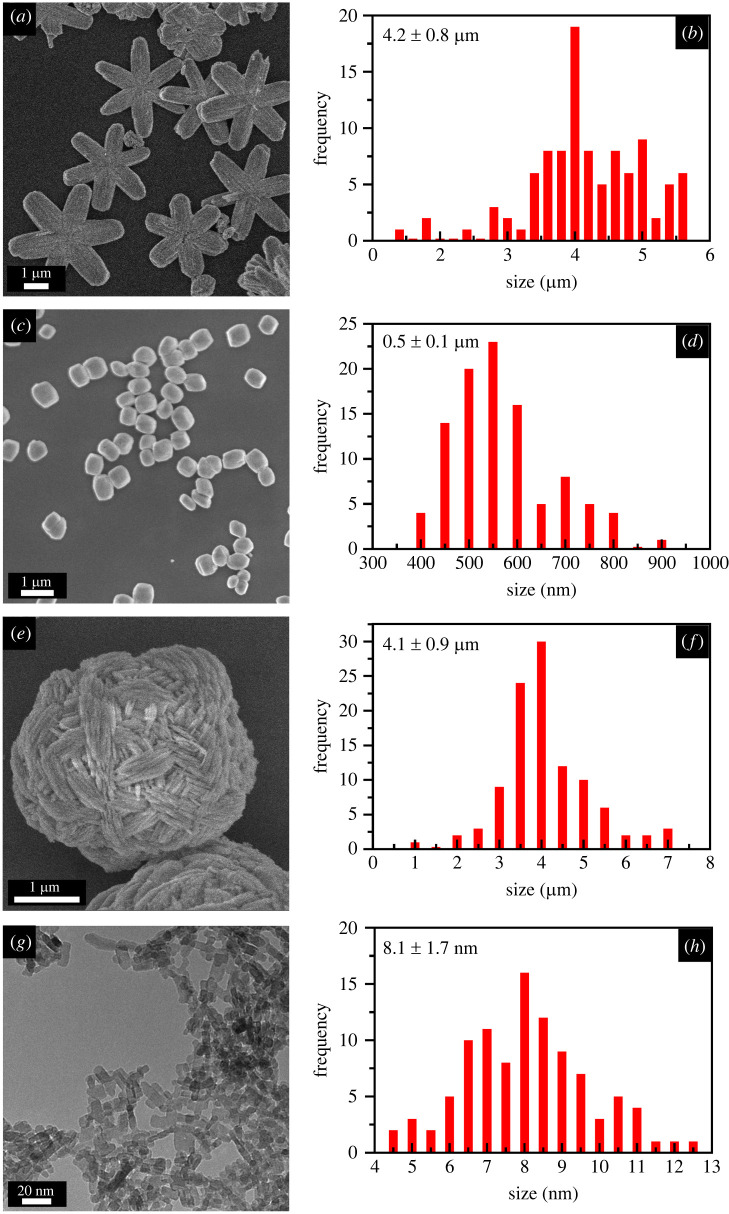
SEM images and size distributions of MgWO_4_ motifs consisting of (*a,b*) stars, (*c*,*d*) large nanoparticles and (*e,f*) wool balls are shown. (*g,h*) TEM images and the associated size distributions are presented for sub-10 nm nanoparticles.

**Figure 2.**
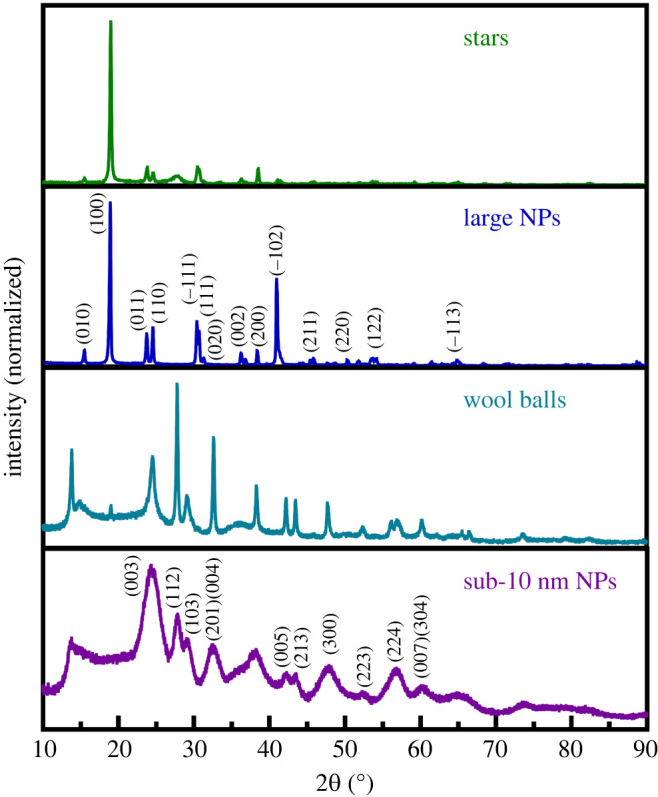
XRD patterns (with indexed peaks) of MgWO_4_ motifs consisting of (from top to bottom): stars, larger nanoparticles, wool balls, and sub-10 nm nanoparticles.

Based on our series of experiments analysing growth conditions, we can highlight the crucial importance of carefully controlling specific reaction parameters, such as surfactant, reaction time, reaction temperature, solvent ratios and pH in inducing the formation of crystallographically pure and monodisperse morphologies of MgWO_4_. Figure 1 highlights SEM and TEM images of different MgWO_4_ particle morphologies that we have prepared. These include (i) ultra-small spherical nanoparticles, (ii) wool balls, (iii) larger spherical nanoparticles, and (iv) stars. Figure 2 represents the XRD pattern of each sample. Both the stars and larger nanoparticles are characterized by a monoclinic phase, whereas the sub-10 nm particles and wool balls can be ascribed to the triclinic phase. The motifs were all synthesized using the hydrothermal method, run at 220°C for 48 h.

### 3.1. Triclinic sub-10 nm particles and wool balls

As depicted in figure 1*g,h*, sub-10 nm particles of MgWO_4_ were produced with a size of 8.1 ± 1.7 nm. Figure 2 is consistent with a pure triclinic phase of MgWO_4_, matching the literature diffractogram that has been previously reported for this material [8]. In terms of accounting for the small sizes of these particles synthesized, we note that pure EG was used as a solvent. EG possesses the ability to form alkoxides with Mg^2+^, and its high viscosity restricts ion migration. As such, Mg^2+^ is less likely to react with ${\mathrm{WO}}_{4}^{2-}$ and hence, larger and discrete nanostructures may not form quite as readily. However, because ultra-small nanoparticles tend to possess relatively low volumes and correspondingly high surface energies [35], they are less stable as individual, isolated and discrete particles, but rather tend to form as larger aggregates as a means of reducing this overall, unfavourable surface energy.

We sought to not only modify the morphology of as-prepared particles but also reduce aggregation by introducing a series of surfactants, such as PVP, PEG, CTAB, SDS and TX-114. The use of surfactants led to either little change in morphology and aggregation state or the introduction of unwanted impurities. The most promising results emanated from the use of PVP. We also observed little to no change in morphology, when the reaction time was varied from 24, 48, to 72 h. Electronic supplementary material, tables S1 and S2 present a summary of all of the trials performed to manufacture sub-10 nm particles, using a variety of different surfactants and solvents. Either increasing or decreasing the reaction temperature apparently had no noticeable effect on these particles.

Wool balls were synthesized using a 3 : 4 EG : H_2_O solvent mixture. Figure 1*e,f* highlights the SEM image and size distribution of the as-prepared wool ball structures. These motifs maintained an average size of 4.1 ± 0.9 µm. A series of experiments was initiated on the wool ball samples to alter their size and shape. As key reaction parameters, we systematically varied the solvent ratios, reaction temperatures and reaction times, respectively.

As we increased the relative amount of EG within the *solvent*, the isolated morphology became less well defined, until no wool balls formed at all (i.e. electronic supplementary material, table S3). This scenario could probably be ascribed to the role of EG in promoting the formation of nanoparticles instead of larger structures. We also tried to alter the morphology by varying the solvent identity to triethylene glycol, ethanol and PEG 400 MW, respectively. These additional trials yielded either (i) primarily impure materials with no distinctive morphology or (ii) samples characterized by heterogeneous mixtures of morphologies. From these collective results, we concluded that the best solvent mixture probably consisted of 3 : 4 EG : H_2_O.

Upon varying *reaction temperature*, we noted that the morphology degraded if the temperature was lower than approximately 200°C (i.e. electronic supplementary material, tables S4 and S5), regardless of the different solvent ratios used. As such, the best reaction temperature we found was 220°C. Finally we varied the reaction time from 1 to 48 h (i.e. electronic supplementary material, table S4). We noted that the isolated morphology and composition did not alter significantly by varying the reaction time. Hence, for the sake of consistency in terms of reaction conditions used for the preparation of analogous samples, we held the reaction time constant at approximately 48 h, unless otherwise stated.

### 3.2. Monoclinic nanoparticles and stars

To produce the larger spherical nanoparticles, we used a 1 : 1 volume mixture of EG : H_2_O in the presence of PVP surfactant and at an overall pH of 10.3. These experimental conditions yielded particles, characterized by relatively large average sizes of 0.5 ± 0.1 µm. Figure 1*c,d* summarizes SEM and associated size distributions for this series of samples. Star-like motifs were isolated when PVP was replaced with PEG 4000 MW. These as-generated stars were characterized by average sizes of 4.2 ± 0.8 µm (figure 1*a,b*). Based on our initial series of experiments, we could state unequivocally that both pH and reaction time appeared to significantly impact the observed morphology and crystallinity, because empirically, we could only isolate the desired monoclinic-phase particles and stars under very specific reaction conditions, implying the need to more systematically vary both pH and reaction time.

**Figure 3.**
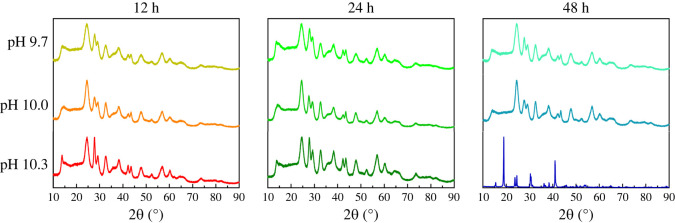
Probing the effects of reaction time and pH. XRD patterns of 1 : 1 EG : H_2_O samples, run under different reaction conditions to produce the desired MgWO_4_ motifs.

Figure 3 presents the XRD patterns of experiments, wherein (i) the reaction time was increased from 12, 24, to 48 h, while simultaneously increasing (ii) the pH from 9.7, 10.0, to 10.3. Electronic supplementary material, table S7 provides the associated SEM images for the samples, prepared during these trials. Based on all of these data, we concluded that samples characterized by a pure monoclinic phase and a well-defined particle morphology could only be isolated at a reaction time of 48 h and at a pH of 10.3. Deviations from these conditions led to the formation of samples, possessing a triclinic crystal structure and/or an imprecise morphology.

### 3.3. Mechanistic insights into the pH effect

To try to account for this pH effect, we note that in essentially all of the prior literature, hydrothermal/solvothermal syntheses of MgWO_4_ are normally conducted at a pH of 9, tuned with the use of 1 M NaOH [8,9,13]. Interestingly, no explanation of why this pH value was chosen was ever given, nor had any systematic investigations of the effect of pH upon the composition and morphology of MgWO_4_ been performed. However, analogous studies on the consequences of varying the pH had indeed been carried out on other similar types of tungstates, including FeWO_4_, ZnWO_4_ and CuWO_4_. In these cases, altering pH apparently had significant ramifications on the observed crystal structure and morphology of the isolated materials [36–39].

As a starting point, it is worth highlighting that in oxides, a crystal phase transition can be induced by a change in pH [37,38]. For example, with BiVO_4_, a monoclinic to tetragonal phase transition was initiated by increasing the pH from 4 to 7; however, this phase transformation was coupled with the introduction of other oxide impurities, theorized to have been created as a result of side reactions between Bi^3+^ and OH^−^ ions [36]. In addition, phase changes for TiO_2_ are well known, with higher pH leading to anatase formation, whereas lower pH yielded rutile [40].

Additionally, other reports on tungstate materials have demonstrated the consequential effects of pH on the corresponding formation of FeWO_4_, ZnWO_4_ and CuWO_4_, respectively. For example, with CuWO_4_, nanospheres, nanorods and nanoplates could be created using pH values of 3, 7 and 10, respectively. Specifically, it was found that increasing pH from 2 to 6 enabled the formation of the CuWO_4_ phase, whereas other pH values tended to produce CuO impurities [41]. Another study on CuWO_4_ noted that lower pH led to the generation of spherical particles, whereas high pH favoured the fabrication of either rods or plates. In particular, the appearance of these anisotropic morphologies was attributed to the presence of an OH^−^ layer, coalescing around the growing particles [39]. With analogous FeWO_4_, hexagonal plates formed at low pH, nanoparticles arose at neutral pH, while nanorods with increasing diameters appeared at higher basic pH values of 9 and 10. The preferential growth of rods at high pH was ascribed to the repulsive electrostatic interactions between OH^−^ and ${\mathrm{WO}}_{4}^{2-}$ species, which resulted in predominant growth along the (002) facets [22,42].

In the case of ZnWO_4_, a wolframite phase was observed at a pH of 9, but Zn(OH)_2_ tended to be the predominant species from pH 9 to 11. Nonetheless, under the high temperature and pressure conditions of the hydrothermal reaction, the Zn(OH)_2_ apparently redissolved [38], and in the process, free ions of Zn^2+^ were able to recombine with ${\mathrm{WO}}_{4}^{2-}$ to form the desired ZnWO_4_. The formation of rods was encouraged by a ‘blocking’ mechanism, enabled by the presence of the OH^−^ ions [38]. In this scenario, the OH^−^ ions were theorized to form an encapsulating layer around the primary nanoparticles, thereby repelling incoming ${\mathrm{WO}}_{4}^{2-}$ ions. This mechanism was responsible for encouraging anisotropic growth along the ends and favouring the generation of nanorods at these high pH values [38].

With MgWO_4_, as mentioned as with the other oxides [36,40], we also detected a phase transition from triclinic to monoclinic upon increasing pH, a finding probably ascribed to the concomitantly higher hydroxide ion concentrations involved. We postulate that side reactions might have occurred between the introduced OH^−^ ions and the Mg^2+^ ions to form Mg(OH)_2_, a white precipitate, that might have been generated during the initial precursor mixing itself, though this species was never actually detected in the XRD patterns of the final products. By analogy with the prior work with ZnWO_4_, we assert that if any Mg(OH)_2_ did form, it subsequently dissolved under hydrothermal conditions and reacted with tungstate ions to produce MgWO_4_ [38].

Therefore, we propose that the MgWO_4_ formed analogously to that of either ZnWO_4_ or FeWO_4_ with respect to the creation of monoclinic stars and nanoparticles. That is, this mechanism would entail that under high pH conditions, OH^−^ ions present at higher concentrations would prevent incoming ${\mathrm{WO}}_{4}^{2-}$ ions from depositing along the sides of the forming crystals and thereby encourage the growth of monoclinic phases. The fact that we were able to produce additional motifs (i.e. nanoparticles and stars), as opposed to nanorods alone under similar reaction conditions, could probably be ascribed to the simultaneous presence of surfactants within the reaction medium and the evolving effects of these additives with pH. That is, in terms of morphology, interactions of OH^−^ with the as-developing MgWO_4_ crystals could potentially induce preferential growth along specific crystalline planes in a synergistic manner with surfactants introduced into solution. In particular, surfactants can bind onto crystal planes and modify their relative surface energy, thereby causing growth along different planes to be either alternately promoted or diminished.

Empirically, we noticed that the presence of the PEG surfactant led to the formation of stars, whereas the use of PVP favoured the generation of large nanoparticles. To explain this difference in behaviour, we analysed the literature to probe the effects of PVP and PEG upon the growth of various analogous tungstate compositions. With respect to PEG, it has been shown to favour the formation of either rod-like or leaf-like morphologies of PbWO_4_. Specifically, it was demonstrated that the PEG molecules bonded selectively through a O-Pb bonding configuration, thereby leading to anisotropic growth along preferred crystal planes [43]. As a different example, in the case of ZnWO_4_, addition of PEG led to the production of smaller nanoparticles as opposed to samples generated in the absence of PEG [44]. With respect to PbWO_4_, the presence of PVP enabled the creation of more monodisperse and isotropic crystals [45]. With NaLa(WO_4_)_2_, micron-scale rod-like shapes were observed at low PVP concentrations, whereas cubes predominated at high PVP concentrations. This scenario probably originated as a result of preferential surfactant attachment onto certain emerging facets at low concentrations, coupled with saturation effects dominating and facilitating the growth of different facets at higher concentrations [46]. Based on these collective results, our MgWO_4_ particles are likely to be stabilized by a coating of PEG molecules, thereby resulting in the fabrication of smaller and relatively uniform spherically shaped nanoparticles. By contrast, the presence of PVP induced the generation of star-like motifs, because of their selective attachment and binding onto other planes.

### 3.4. Optical properties

#### 3.4.1. Photoluminescence

Photoluminescence data on our MgWO_4_ were obtained to probe the effects of changing the crystal structure and morphology upon the resulting optical properties of our samples. First, we considered and compared the two distinctive crystal structures, using different excitation wavelengths. Since our samples consisted of two crystal phases, namely triclinic and monoclinic, we used an annealing method to convert the triclinic wool balls and sub-10 nm particles to their monoclinic phase counterparts, in order to better compare the properties of these different samples. Specifically, annealing the wool balls and sub-10 nm particles at 800°C for 3 h allowed for a transition from the initial triclinic phase to the desired monoclinic phase. Electronic supplementary material, figure S1 highlights the images and associated size distributions for both of the annealed samples. We found that the wool balls maintained not only a ball-like shape but also a size range of 4.0 ± 0.6 µm, which was within the standard deviation of the dimensions of the original unannealed particles. However, the smaller, individual constituent components of the wool-ball spherical motifs appeared to have fused together to yield smoother and larger particles. Annealing of these sub-10 nm particles led to a significant increase in particle size from 8.1 ± 1.7 to 29 ± 9.9 nm, indicative not only of larger particle formation but also of an increase in size polydispersity. Nonetheless, both samples of wool balls and particles were characterized by a monoclinic crystal structure; electronic supplementary material, figure S2 shows the corroborating XRD spectra of these annealed samples.

**Figure 4.**
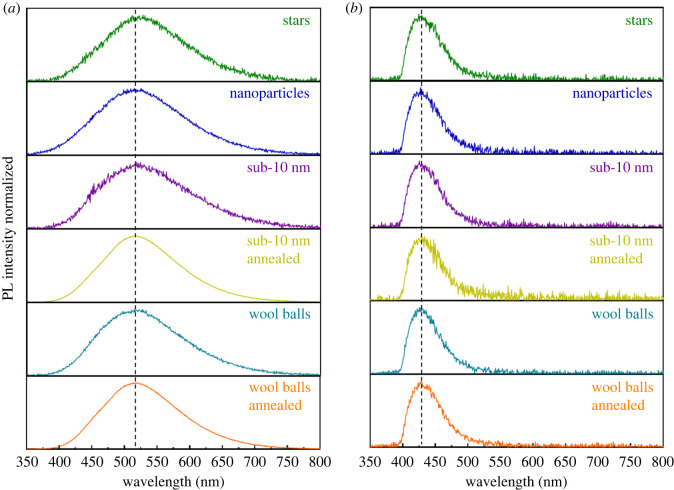
PL emission plots for all samples of MgWO_4_, (*a*) excited at 280 nm with an emission maximum located at 515 nm and (*b*) excited at 340 nm with an emission maximum at 421 nm. The positions of the emission maxima are indicated by the black dashed lines.

Based on prior literature, triclinic samples tend to fluoresce when exposed to 340 nm excitation, whereas the monoclinic phase fluoresces upon excitation with a 280 nm laser [18]. As such, we initially chose these excitation wavelengths to study the materials. Figure 4*a* and *b* highlight the emission profiles of all six of the analysed samples, upon excitation at 280 and 340 nm, respectively. As mentioned earlier, the triclinic samples incorporated two distinctive morphologies, namely sub-10 nm particles and micron-scaled wool balls. Upon excitation at 340 nm, we observed a single emission maximum, located at 421 nm (figure 4*b*).

**Figure 5.**
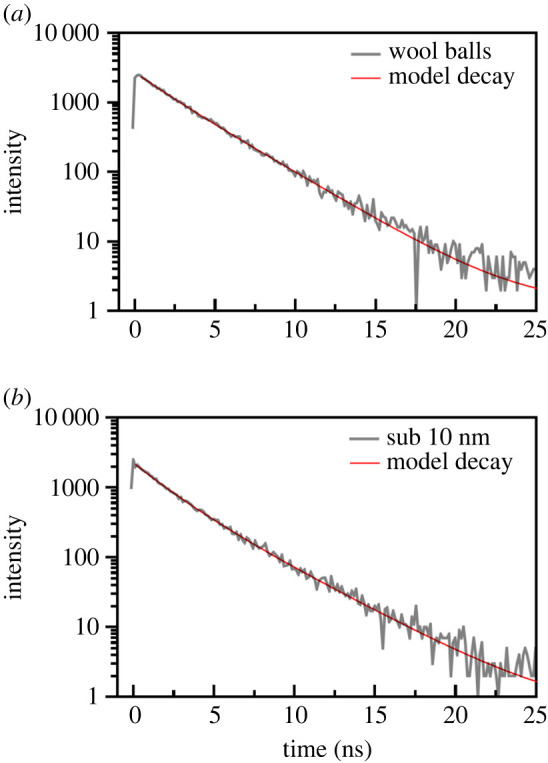
PL decay plots for the unannealed triclinic samples, observed for (*a*) wool balls and (*b*) sub-10 nm particles. The excitation was 340 nm and the PL decay was measured at the 421 nm emission peak.

The PL decay times for these triclinic wool balls and sub-10 nm particles were approximately 3 ns; figure 5 highlights the associated plots of PL decay. These data were consistent with the previous literature results reported for triclinic samples. In particular, the observed luminescence was attributed to not only surface defect sites but also oxygen vacancies in the amorphous MgWO_4_ [18]. Upon irradiation with the 340 nm laser, the monoclinic phase samples (including annealed wool balls, annealed sub-10 nm particles, stars and nanoparticles) also unexpectedly yielded a peak situated at approximately 420 nm. This finding suggests that surface defects are probably still present on the external surfaces of this monoclinic phase sample. It should be noted that with these samples, there is a fairly significant level of noise in all of the collected spectra, indicative of a poor emission signal at this excitation wavelength.

In parallel experiments in which all samples were excited by 280 nm (figure 4*a*), the PL spectra of as-generated monoclinic samples were characterized by a single, discrete signal localized at 515 nm. Indeed, literature was consistent with the presence of a single peak for monoclinic MgWO_4_, located at approximately 470 nm, which could be ascribed to the presence of both (WO_6_)^6−^ octahedra and defect-induced (WO_4_)^2−^ tetrahedra [18]. Nevertheless, our samples exhibited a notable red shift of approximately 45 nm, as compared with the data from the prior literature. This apparent red shift was observed for all of our monoclinic samples. Indeed, for these materials, the observed PL decay was dominated by a long component of the order of approximately 7.0 µs. Electronic supplementary material, figure S3 illustrates the PL decay plots, obtained at 515 nm [18]. Similarly, the triclinic-phase samples all displayed this emission peak at 515 nm.

To better understand the nature of the emission within the samples, we collected a set of excitation spectra, presented in electronic supplementary material, figure S4. These spectra indicate two distinctive patterns with respect to the monoclinic- and triclinic-phase samples. The monoclinic-phase sample possesses a very strong excitation maximum at 296 nm, whereas the triclinic samples only give rise to a weak excitation in this region. In particular, the excitation spectra indicate a clear dependence upon shape and the relative intensity of the components below 300 nm. There is also no notable excitation at 340 nm for all of these samples, despite the 421 nm emission observed when this excitation wavelength was used. The lack of an excitation peak at 340 nm in the excitation spectra may be due to the relatively low intensity of the 420 nm luminescence. Additionally, the PL emission around 420 nm might have been caused by using a fs pulsed laser with a wavelength of 340 nm, which has a very high energy associated with each pulse. The emitting centre may be inefficiently excited with the Xe lamp and the monochromator used for the excitation measurement, thereby resulting in it not being readily detected. This excitation maximum is also red-shifted by approximately 45 nm from the 470 nm reported in the prior literature [18] as compared with the 515 nm position reported in this work.

#### 3.4.2. Radioluminescence

**Figure 6.**
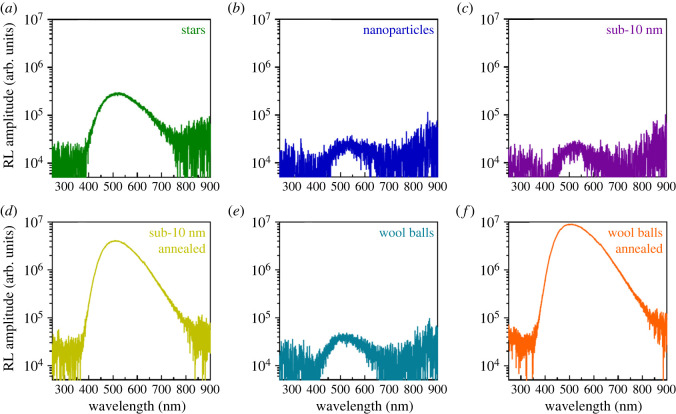
RL emission plots for samples of MgWO_4_ with different morphologies and crystal structures, including (*a*) stars, (*b*) larger nanoparticles, (*c*) sub-10 nm particles (unannealed), (*d*) sub-10 nm particles (annealed), (*e*) wool balls (unannealed), and (*f*) wool balls (annealed). All graphs are displayed in a log10 scale for easier comparison.

In terms of radioluminescence, all samples evince similar spectra with a broad emission, centred at approximately 500 nm (figure 6). Prior literature of a bulk MgWO_4_ scintillator was indicative of a single broad emission maximum, positioned at 470 nm. Interestingly, this RL emission result is in line with our reported positions of the PL peaks. However, with respect to our as-prepared samples, we observed a notable red shift of approximately 30 nm as compared with that reported for the bulk in earlier studies. There seemed to be also a slight though perceptible variation in the shape of the spectra, especially at the long wavelengths. However, this observation may have been due to the comparatively low signal of the monoclinic-phase nanoparticles, triclinic-phase wool balls and triclinic-phase sub-10 nm particles (figure 6*b*,*c*, and *e*). By contrast, the monoclinic-phase stars gave rise to a much higher (approx. an order of magnitude greater) level of emission (figure 6*a*).

We note that the RL intensity strongly depended on the sample processing treatment, with unannealed sub-10 nm nanoparticles and ‘wool-ball’ shapes yielding the lowest signal intensity. After annealing though, we observed a significant increase in the RL intensity by nearly two orders of magnitude. Table 1 summarizes the light output values, estimated by comparing the observed intensity with that of a YAlO_3_: Ce standard. Based on this comparison, the unannealed wool balls and sub-10 nm particles maintained emissions of 0.30% and 0.19%, whereas the corresponding annealed wool balls and annealed sub-10 nm particles gave rise to a signal equivalent to 54% and 25% of YAlO_3_: Ce, respectively. These data highlight an increase of approximately 180× for the wool balls and approximately 131× for the sub-10 nm particles, respectively, as compared with control samples.

**Table 1.** RL light yield for as-synthesized particles, tested herein. Light yields were estimated by comparing the measured output with that of a YAlO_3_: Ce standard.

sample	light output (photons (MeV)^−1^)	relative light output (%)	Scherrer size (nm)
YAlO_3_: Ce Standard	24 341	100.00	N/A
stars	637	2.62	25.17
larger nanoparticles	44	0.18	32.11
sub-10 nm (unannealed)	47	0.19	5.17
sub-10 nm (annealed)	6120	25.14	36.67
wool balls (unannealed)	74	0.30	26.40
wool balls (annealed)	13 163	54.08	41.41

**Figure 7.**
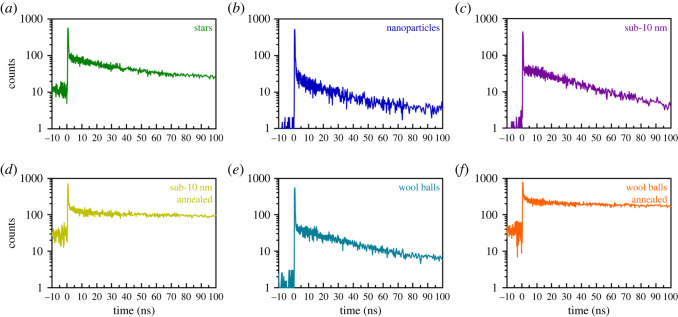
RL time decay plots for samples of MgWO_4_ characterized by different morphologies and crystal structures, including (*a*) stars, (*b*) larger nanoparticles, (*c*) sub-10 nm particles (unannealed), (*d*) sub-10 nm particles (annealed), (*e*) wool balls (unannealed) and (*f*) wool balls (annealed).

Figure 7 provides for RL decay plots for all samples; a three-exponential fit was used to calculate the decay times, provided in table 2. In terms of the associated measured RL time decays, all samples displayed a very fast component situated at approximately 0.2 ns that accounted for a relatively small amount of the decay. The monoclinic stars, nanoparticles, sub-10 nm particles and wool balls gave rise to comparatively similar behaviour incorporating both a fast and slow decay component. The fast component was calculated to be 38.7, 32.5, 36.4 and 38.6 ns for the monoclinic stars, nanoparticles, sub-10 nm particles and wool balls, respectively. The corresponding slow component for these samples was computed to be 2.05, 1.23, 1.24 and 1.30 µs, respectively. On the other hand, the behaviour of the annealed sub-10 nm nanoparticles and annealed wool ball samples was almost solely dominated by a slow component of the order of 4.23 and 4.90 µs, respectively. These measured values, which were several times faster than the long decay times previously established from the prior literature for bulk MgWO_4_ scintillators, were characterized by decay times of 36 µs [5]. It needs to be mentioned that the flat background, representing a large portion of the overall signal (figure 7) obtained on all samples, is significantly higher than that of the instrumental background. This anomalously much higher signal may very well be related to the presence of longer scintillation decay components, whose measured decay is not appreciable within the range of the reported time scales.

**Table 2.** RL time decay data for the as-synthesized particles. All samples were fitted with a three-exponential fit. The relevant results are indicated by Tau 1, 2, and 3. A1, 2, and 3 refer to the relative fraction (as an area) of the decay, while C is associated with the remaining background. These values are reported out of 100%.

sample	Tau 1 (ns)	A1	Tau 2 (ns)	A2	Tau 3 (ns)	A3	C
stars	0.16	0.2	38.7	2.7	2056	23	74
larger nanoparticles	0.12	3	32.5	9	1227	25	63
sub-10 nm (unannealed)	0.1	3	36.4	22	1243	27	48
sub-10 nm (annealed)	0.2	<0.1	99	0.4	4235	25	75
wool balls (unannealed)	0.1	4	38.6	12	1301	33	51
wool balls (annealed)	0.3	<0.1	161	0.5	4903	27	62

Based on previous literature reports, it had been shown that the emission of the triclinic phase MgWO_4_ can be attributed to the presence of surface defects, such as the oxygen vacancies, of more amorphous materials. It has also been demonstrated that an increase in annealing temperature can lead to a decrease in oxygen vacancies and related defects [18]. Based on this finding and the previously reported changes in crystal structure before and after annealing, we calculated the crystallite size of our samples from the Scherrer equation, based on XRD data, to gain a better understanding of the associated changes in crystallinity, which might explain our RL data. Results of these calculations are presented in table 1.

With respect to the sub-10 nm particles, we noted an increase in the crystallite size of the sample motif from 5.17 to 36.67 nm after annealing. Similarly, the crystallite size of the wool balls analogously increased from 26.40 to 41.41 nm upon annealing. Both of the observed increases in crystallite size were coincident with the rise in RL emission noted for the annealed samples. We also found that the calculated crystallite size of the annealed samples is also larger than that of any of the hydrothermally generated, monoclinic samples, including stars and larger nanoparticles. Furthermore, the low RL intensity of the unannealed sample may be ascribed in part to impurity-induced partial quenching, that has been found to occur at room temperature in MgWO_4_ crystals [47]. As such, we propose that the high RL intensity of the annealed samples may be a result of the higher sample crystallinity and consequentially, greater sample purity, due to the likely removal of luminescent-quenching defects during the thermal heating process.

### 3.5. Cellular uptake and toxicity

To test the viability of our MgWO_4_ samples as potential biocompatible platforms, we needed to not only demonstrate their successful uptake by various cells but also assess their relative toxicity with respect to active, functional biological systems. As such, to prepare the particles for imaging and cell uptake, the particles were coated with a layer of APTES, which binds to the surface and provides for free amine groups that can allow for the relatively facile attachment of standard biologically compatible fluorescence dyes, namely FITC in our experiments. Finally, PEG was added to improve the uptake of the particles into the cells and to minimize possible aggregation effects.

**Figure 8.**
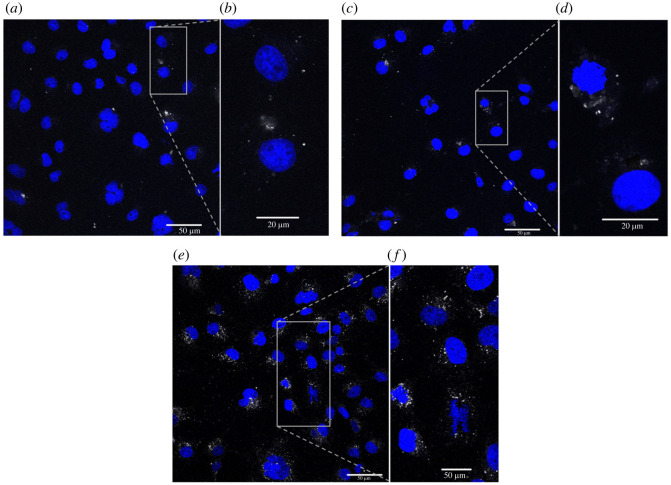
Confocal images of COS7 cells (nuclei labelled blue), demonstrating uptake of FITC-labelled particles (in white). (*a*) Confocal image of COS7 cells incubated with particles for 2 h. (*b*) Higher magnification image of cells in (*a*), illustrating a more detailed view. (*c*) Confocal image of COS7 cells incubated with particles for 12 h. (*d*) Higher magnification image of cells in (*c*), highlighting a more detailed view. (*e*) Confocal image of COS7 cells incubated with particles for 18 h. (*f*) Higher magnification image of cells in (*e*), showing a view given in greater detail.

The excitation wavelength of the sample particles was between 280–340 nm, as discussed in §3.4.1. This excitation value lies outside the range available on most confocal microscopes, meaning that we could not realistically use the intrinsic fluorescence of these oxides as an effective label. That is, the shortest wavelengths that can be probed with the majority of confocal microscopes is 405 nm. As such, it was necessary to attach a different and more appropriate label (i.e. FITC) to successfully visualize the particles within the cells. The final material tested therefore consisted of MgWO_4_-APTES-FITC-PEG particles, which were taken up by COS and HEK cell lines starting within 2 h, with the measured uptake found to increase over time. We detected the relative amount of uptake by means of tracking FITC emission using confocal imaging, as shown in figure 8.

**Figure 9.**
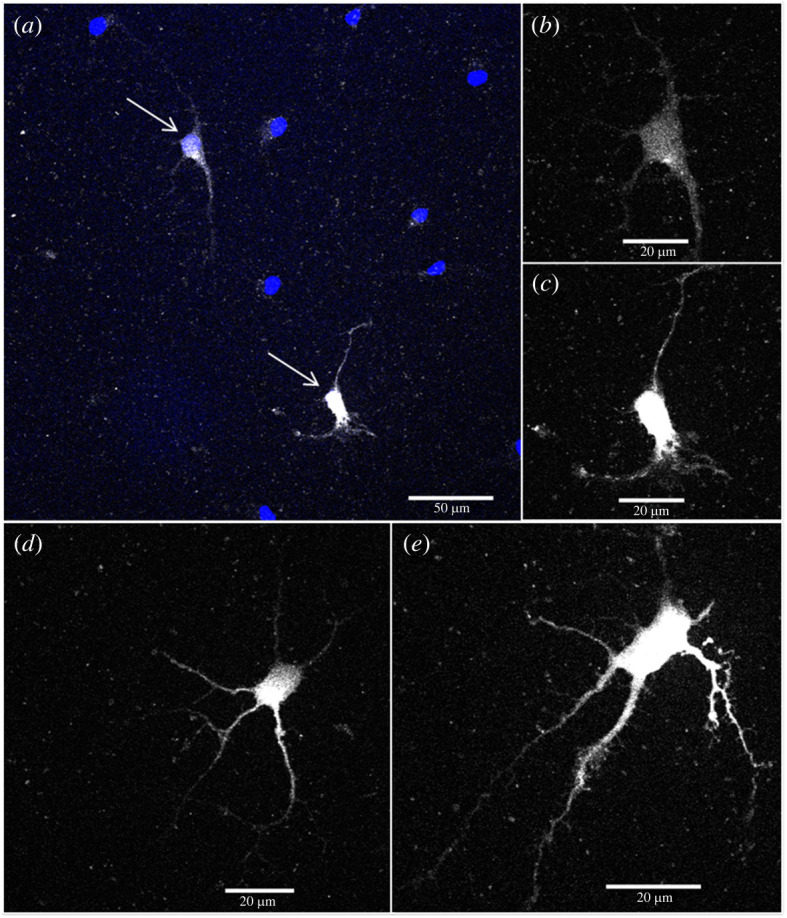
Confocal images of neurons (nuclei labelled blue), showing the uptake of FITC-labelled particles (in white). (*a*) Confocal image of neurons incubated with particles for 20 h. Arrows point to the positions of neurons, shown in (*b*) and (*c*). (*b,c*) Higher magnification images (FITC channel only) of selected neurons, providing details of the observed particle uptake in axons/dendrites. (*d,e*) Additional images of neurons (FITC channel only), demonstrating particle uptake coupled with details of associated axons/dendrites.

We noticed a diffuse signal of particle fluorescence consistent with a possible freely available particle presence in the cytoplasm. Nevertheless, there was also particle signal appearing to be concentrated into points, due to aggregation of the particles and/or their accumulation within endosomes/lysosomes after cellular uptake. Particles were clearly taken up by both COS and HEK cells. Importantly, robust particle uptake was detected in primary dissociated embryonic hippocampal neurons. Of particular interest is that in these cultured neurons, the particles were clearly present in the neuronal soma and in the axons and dendrites, whereby the axonal/dendritic processes were rendered clearly visible, as shown in figure 9. Further development of this coating methodology, beyond the scope of the current study, could reduce the level of aggregation and improve the consistency of cellular uptake.

**Figure 10.**
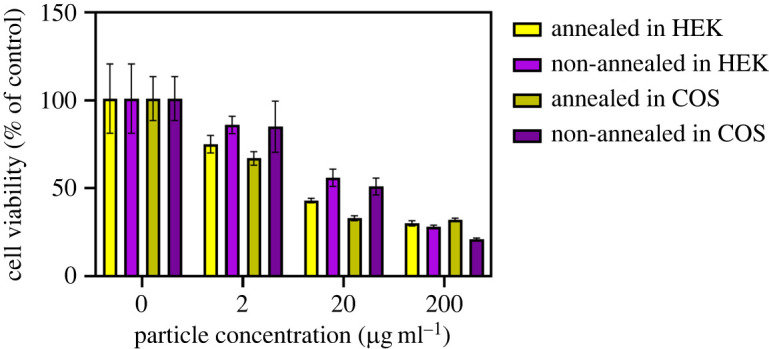
Cell viability assays, taken using MTT in both HEK and COS cell lines, incubated with annealed and non-annealed, sub-10 nm particles. Different concentrations of MgWO_4_ particles were probed, including 0 μg ml^−1^ for the control sample, followed by 2, 20 and 200 μg ml^−1^ concentrations, respectively, of the relevant oxide particle samples.

As observed from figure 10, cell viabilities, as deduced from the MTT assay prepared at a dosage of 2 µg ml^−1^, ranged from 67% to 84%, while at a dosage of 20 µg ml^−1^, these quantities spanned an interval from 33% to 54%. At the highest dosage value of 200 µg ml^−1^, they varied from 21% to 32%. From these data, we noted an expected increase in toxicity with increasing dosage. A perceptible decrease in cell viability at the 2 µg ml^−1^ dosage suggests some degree of inherent toxicity. Enabling a reduction in cellular toxicity, perhaps using the well-studied method of coating the particles with silica, would be an area of further analysis of this system. Furthermore, we are encouraged by (i) the robust particle uptake by cultured primary hippocampal neurons and their stable presence in these neurons for a prolonged time (approx. 20 h) and (ii) the apparent survival and intact development of the axons and dendrites, all of which are highly indicative of low particle toxicity, since developing primary neurons have low tolerance and undergo rapid apoptosis, following induction of many types of stressors.

## 4. Conclusion

We have successfully synthesized four distinctive morphologies of particulate motifs, including stars, larger nanoparticles, wool balls and sub-10 nm particles of MgWO_4_. We utilized a ‘one-pot’ hydrothermal method to generate these particles and demonstrated the consequences of changing a variety of variables, including but not limited to reaction time, solvent ratios, temperature, surfactants and pH, on the resulting crystal structure and morphology. We attributed (i) crystal structure control to the ‘blocking’ effect of OH^−^ ions at high pH and (ii) morphology control to the use of mixed solvents and surfactants. Variables, such as reaction time, pH and reaction temperature, all needed to be kept constant to synthesize stars and large nanoparticles, characterized by the monoclinic phase. Additionally, we have demonstrated the ability to reliably change the crystal structure of the wool balls and sub-10 nm particles. Prior to annealing, the wool balls and sub-10 nm samples maintained a triclinic-phase crystal structure. Heat treatment at 800°C led to a complete phase change from the triclinic to the monoclinic phase, as suggested by XRD.

With respect to the optical properties, we measured PL data for the monoclinic- and triclinic-phase samples with emission maxima noted at 515 nm upon excitation at 280 nm. The collected RL data indicated the importance and prominence of the thermal annealing process (as opposed to other factors, such as the intrinsic morphology or size of the oxide samples themselves) on these materials in order to obtain the highest emission signal possible. Specifically, the series of annealed wool balls and annealed sub-10 nm particles displayed greater than two orders of magnitude higher emission as compared with their unannealed counterparts.

We have also demonstrated the ability to functionalize MgWO_4_ particles with a biocompatible coating and to have them taken up by neurons without dramatically reducing cell viability, which forms the basis for the promising use of these materials in biomedical applications. Hence, based on the combination of cellular uptake data, indicative of their apparent biocompatibility, coupled with the broad radioluminescence emission centred at 500 nm, these collective results suggest that these readily formed metal oxide nanomaterials represent excellent candidates for PDT. Moreover, we have noted that several compatible photosensitizers could potentially activate the radioluminescence emission; possible candidates include but are not limited to merocyanine-540 [48] and Rose Bengal [49], which both maintain excitations at approximately 550 nm. Further study beyond the scope of the current work will be needed to realize that enormous potential and to demonstrate the effective utility of these particles for relevant applications, such as but not limited to photodynamic therapy.

## Data Availability

All research materials supporting the data and conclusions described in the manuscript have been provided either within the main text itself or in the 19 pages of the Supporting Information section, associated with the final published version of the manuscript. The data are provided in electronic supplementary material [50].
